# Cardiac index and oxygen delivery during low and high tidal volume ventilation strategies in patients with acute respiratory distress syndrome: a crossover randomized clinical trial

**DOI:** 10.1186/cc12825

**Published:** 2013-07-23

**Authors:** Giuseppe Natalini, Cosetta Minelli, Antonio Rosano, Pierluigi Ferretti, Carmine R Militano, Carlo De Feo, Achille Bernardini

**Affiliations:** 1 General Intensive Care Unit, Poliambulanza Foundation Hospital, Brescia, Italy; 2 Centre for Biomedicine, EURAC research, Bolzano, Italy; 3 Intensive Care Unit, Desenzano del Garda Hospital, Desenzano del Garda, Italy

**Keywords:** Cardiac Output, Tidal Volume, Respiratory Distress Syndrome, Adult, Hemodynamics

## Abstract

**Introduction:**

The beneficial effect of low tidal volume (TV) ventilation strategy on mortality in patients with acute respiratory distress syndrome (ARDS) has been attributed to the protective effect on ventilator-induced lung injury, and yet its effect on cardiovascular function might also play an important role. The aim of this study was to assess whether low TV ventilation improves cardiac output and oxygen delivery compared with high TV ventilation strategy in patients with ARDS.

**Methods:**

In this crossover randomized clinical trial 16 ARDS patients were recruited in an intensive care unit at a university-affiliated hospital. Each patient was ventilated for 30 min with low (6 mL/kg) and 30 min with high (12 mL/kg) TV. The two experimental periods, applied in random order and with allocation concealment, were separated by 30 min of basal ventilation. Minute ventilation was constantly maintained by appropriate respiratory rate changes.

**Results:**

Compared with high TV ventilation, low TV ventilation showed decreased pH (7.37 *vs*. 7.41, *P *= 0.001) and increased PaCO_2 _(49 *vs*. 43 mmHg; *P *= 0.002). Cardiac index and oxygen delivery index were increased with low compared with high TV ventilation (3.9 *vs*. 3.5 L.min^-1^.m^-2^, *P *= 0.012, and 521 *vs*. 463 mL.min^-1^.m^-2^, *P *= 0.002, respectively), while oxygen extraction ratio decreased (0.36 *vs*. 0.44, *P *= 0.027). In four patients oxygen extraction ratio was >0.5 during high TV but not during low TV strategy. The magnitude of the change in cardiac index was positively associated with PaCO_2 _variation (*P *= 0.004), while it was unrelated to the magnitude of changes in TV and airway pressure. The decrease of cardiac index was predicted by PaCO_2 _reduction, with and area under ROC curve of 0.72.

**Conclusions:**

Our findings suggest that a low TV ventilation strategy increases cardiac index and oxygen delivery, thus supporting the hypothesis that the beneficial effect of low TV ventilation in patients with ARDS could be partially explained by hemodynamic improvement. In other words, low tidal volume ventilation could be protective also for the cardiovascular system and not only for the lung. The slight increase of PaCO_2 _during low TV ventilation seems to predict the increase of cardiac index.

**Trial registration:**

ClinicalTrials.gov: NCT00713713

## Introduction

Low tidal volume (TV) decreases mortality in patients with acute respiratory distress syndrome (ARDS) as opposed to high TV ventilation [1]. There is convincing evidence that low TV reduces lung and systemic inflammatory response compared to high TV ventilation [2]. This effect is attributed to a decrease in lung stress and strain [3] and alveolar cyclic recruitment-derecruitment [4] that is considered the main factors triggering ventilator-induced lung injury.

The impact of low TV ventilation on the complex coupling between respiratory and cardiocirculatory function might also contribute to its observed beneficial effect. Cardiac output can be modified by tidal inspiration and end-expiratory lung volume through both lung volume change and preload dependence of stroke volume [5,6]. Moreover tidal increase in lung volume impacts cardiac output in different ways if ventilation is driven by spontaneous activity or by positive pressure ventilation [7,8]. The interaction between all these variables can have different results depending on the mechanical properties of lung and chest wall, and for these reasons clinical studies on heart-lung interaction are best carried out in homogeneous groups of patients with well-defined cardiovascular and ventilatory patterns.

Previous clinical studies investigated the effect of ventilation with different TVs on cardiac output in ARDS patients [9-13]. Nevertheless these investigations were carried out before current concept of protective low TV ventilation that is based on the use of TV 6 mL/kg of ideal body weight in assist-control mode [1] and they tested different TVs in deeply sedated and paralyzed patients. Therefore their results were conflicting and difficult to apply to current clinical practice.

To the best of our knowledge, there is only one study on ARDS patients which compared the cardiovascular effect of TV strategies used in ARDS Network trial [14]. This study did not show any difference in arterial blood pressure and heart rate between different TV strategies but unfortunately cardiac output was not measured.

We hypothesized that if low *versus *high TV strategy increases cardiac output and oxygen delivery, we could explain the protective effect of low TV not only in terms of prevention of ventilator-induced lung injury but also with the improvement in cardiovascular function.

The aim of the present study was to assess whether low TV improves cardiac output and oxygen delivery compared to high TV strategy in ARDS patients during assisted control ventilation with constant minute ventilation.

## Materials and methods

The protocol was approved by the institutional ethical committee (Comitato Etico delle Istituzioni Ospedaliere Cattoliche) and written consent had to be obtained from the patients or their next of kin if the patients themselves were not competent.

We performed a sample size calculation assuming a mean baseline cardiac index of 4.3 L.min^-1^.m^-2^, with standard deviation of 1.4, as described for ARDS patients by Kondili and colleagues (15). We calculated that 16 patients were needed to detect a within-patient difference in cardiac index of 25% for low *versus *high TV strategy, with power of 0.80 and level of significance of 0.05. We therefore recruited 16 consecutive patients admitted to the Intensive Care Unit (ICU) of Poliambulanza Foundation Hospital who satisfied all of the following criteria: diagnosis of ALI/ARDS according to American-European Consensus Conference criteria [16]; age >18 years; and monitoring of cardiac output. Patients were excluded if they had mean arterial pressure <65 mmHg or if the infusion rate of vasoactive agents had been modified in the 2 h before study enrollment.

All patients were ventilated using pressure controlled ventilation with volume target (Pressure-Regulated Volume-Control Ventilation, Siemens-Elema Servo 300, Solna, Sweden). The ventilator setting at enrollment was defined as basal ventilation. Sedative drugs were targeted to obtain Richmond Agitation Sedation Score 0 or -1 and spontaneous respiratory activity was allowed when patient and ventilator respiratory phases were synchronous as judged by visual inspection of airway pressure and airflow waveforms. Deep sedation (with or without muscle paralysis) was reserved for cases of refractory patient-ventilator asynchrony or severe hypoxemia. Deep sedation was obtained with continuous infusion of propofol titrated to obtain no response to voice or physical stimulation (Richmond Agitation-Sedation Score -5). Cisatracurium besilate 0.2 mg/kg was administered if patient-ventilator asynchrony was persistent despite deep sedation.

Patients' characteristics are shown in Table 1.

**Table 1:** Patients' characteristics.

Body mass index (kg/m^2^)	28 (7)
Body surface area (m^2^)	1.87 (0.27)
Age (years)	60 (14)
Sex (female)	8 (50%)
Main diagnosis at ICU admission:	
Pneumonia	5 (31%)
Multiple trauma	3 (19%)
Brain injury	2 (12%)
Extrapulmonary sepsis	6 (38%)
Pulmonary ARDS	10 (63%)
Patients with vasoactive agents during the study	12 (75%)
Hemoglobin (g/dL)	9.9 (1.9)
PaO_2_/F_I_O_2 _(mmHg)	192 (61)
Positive end-respiratory pressure (cmH_2_O)	14 (3)
F_I_O_2_	0.64 (0.14)
Hospital survival	10 (63%)

Data are shown as mean (standard deviation) or count (%).

ARDS: acute respiratory distress syndrome.

### Study design

We used a crossover randomized trial design, with each patient ventilated 30 min with low and 30 min with high TV strategy. The two experimental periods were applied in random order according to a computer generated randomization list, and they were separated by 30 min of basal ventilation. The sequence of the two TV strategies was visible only after the enrollment to assure allocation concealment.

Positive end-expiratory pressure, inspiratory oxygen fraction, minute ventilation, and duty cycle remained constant throughout the study, while respiratory rate was modified to maintain constant minute ventilation.

#### Low TV strategy

TV was set at 6 mL/kg of ideal body weight and respiratory rate could be modified to maintain minute ventilation at the same level as during basal ventilation. Plateau pressure was limited to 30 cmH_2_O in patients without signs of spontaneous respiratory activity during 3-s end-inspiratory pauses. Alternatively peak airway pressure was limited to 35 cmH_2_O when stable plateau pressure was not obtained because of patient respiratory activity.

#### High TV strategy

TV was set at 12 mL/kg of ideal body weight and respiratory rate was modified to maintain minute ventilation at the same level as during basal ventilation. Plateau pressure was limited to 45 cmH_2_O in patients with plateau pressure during a 3-s end-inspiratory pause. Alternatively peak airway pressure was limited to 50 cmH_2_O.

The infusion rate of vasoactive and sedative drugs was not modified during the entire study period.

### Study outcomes

The primary outcome was the difference in cardiac index measured during low and high TV strategy. Secondary outcomes were differences in oxygen delivery, oxygen consumption, and central venous saturation with low and high TV strategy.

### Measurements

Cardiovascular and respiratory variables were recorded during basal ventilation (immediately before the beginning of both low and high TV periods) and during low and high TV ventilation (at the end of each study period).

Airway pressure and flow were measured at the airway opening, arterial blood pressure, heart rate, and central venous pressure were continuously monitored via arterial and central venous catheters, and oxygen consumption was measured by indirect calorimetry (Datex-Engstrom CS/3 Critical Care Monitor, Datex-Engstrom Division, Instrumentarium, Helsinki, Finland).

Heart rate, mean blood arterial pressure, central venous pressure, peak and mean airway pressure, TV, respiratory rate, carbon dioxide production, and oxygen consumption were all measured every 10 s and recorded for 5 min on a personal computer using a specific software (Datex-Ohmeda S/5 Collect, Datex-Ohmeda Division, Instrumentarium Corp., Helsinki, Finland), with their mean value being used for analysis. Systemic vascular resistance and oxygen extraction ratio were calculated with standard formulas.

Arterial and central venous blood samples were obtained for blood gases, and lactate concentration was measured from arterial blood samples (AVL OMNI 1-9 Modular System, AVL LIST Medizintechnik, Graz, Austria).

Cardiac output was obtained by pulmonary artery catheter (Edwards Lifesciences LLC, Irvine, CA, USA) or Pulse-induced Continuous Cardiac Output (PiCCO, Pulsion Medical System AG, Muenchen, Germany) depending on the device used for clinical management. The correct placement of the pulmonary artery catheter was confirmed by the appropriate pressure traces on insertion and by chest radiography. Cardiac output measurement was always obtained by thermodilution with both devices, three consecutive measurements within 10% were required, and their mean value was used for analysis. We only analyzed hemodynamic variables that could be obtained both from pulmonary artery catheter and PiCCO: cardiac output, central venous pressure and saturation, systemic vascular resistance.

The ratio of physiologic dead space to TV was calculated as previously described [11]. The difference between actual and set minute ventilation was interpreted as the contribution of spontaneous respiratory activity to minute ventilation, and it was expressed as percentage of minute ventilation.

### Statistical analyses

Data are shown as mean (standard deviation), median (interquartile range, IQR), and count (percentage), as appropriate. Differences between the two TV strategies for the parameters of interest were tested using an analysis of variance adjusted for their baseline value (value of the parameter before application of each ventilation strategy), with the model thus containing a term for patient, ventilation strategy, and baseline value. We also tested for the presence of a period effect and a period*ventilation interaction (carry-over) effect by adding them into the model. Variables that were not normally distributed were log transformed before analysis. Relationships between continuous variables were analyzed by linear regression. Predictors of cardiac index increase from high to low TV strategy were evaluated using the area under the receiver operating characteristic (ROC) curve, and sensitivity and specificity were calculated for variables which had an area under the ROC curve >0.7. Statistical analyses were performed using R statistical software (R Foundation for Statistical Computing, Vienna, Austria).

## Results

**Figure 1:**
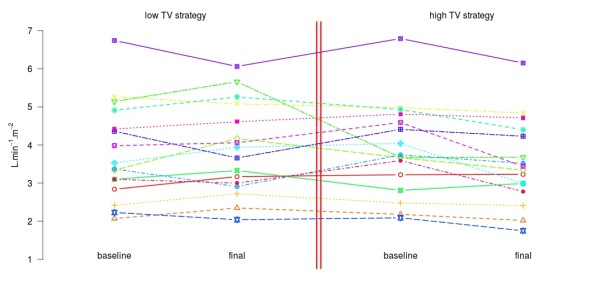
**Values of cardiac index throughout the study for each individual, with baseline and final values for low and high tidal volume (TV) ventilation strategy**. In the study, the sequence of low TV and high TV ventilation was randomized, so that the figure does not provide any information about the actual order in which the two ventilation strategies were applied.

Nine out of 16 patients (56%) showed signs of spontaneous respiratory activity synchronous with mechanical ventilation at enrollment in the study, and seven patients were deeply sedated. Among patients with spontaneous respiratory activity at baseline, all of them maintained it during low TV ventilation, while six of them became passive during high TV ventilation. All patients without spontaneous respiratory activity at enrollment were deeply sedated, and they did not show any respiratory activity throughout the study. Cardiac index was measured in 11 patients (69%) with a pulmonary artery catheter and in five (31%) with a PiCCO. Values of cardiac index throughout the study for each individual patient are shown in Figure 1.

Basal TV, peak and mean airway pressure, blood gases, and cardiovascular variables were similar before low and high TV strategies were applied (Table 2), assuring that the two experimental ventilation strategies started from similar conditions.

**Table 2:** For all parameters, reported are the value before ('Baseline') and after ('Final') each ventilation strategy, summarized as mean (standard deviation).

Parameter	Low VT strategy		High VT strategy		Low *vs*. high TV	
	**Baseline**	**Final**	**Baseline**	**Final**	**Difference *P *value**	

Tidal volume (L)	0.49 (0.1)	0.42 (0.07)	0.50 (0.1)	0.72 (0.18)	-0.30	***<0.001***
Tidal volume/ideal body weight (mL/kg)	8 (2)	7 (1)	8 (2)	12 (3)	-5	***<0.001***
Respiratory rate (1/min)	22 (3)	25 (5)	22 (5)	15 (5)	10	***<0.001***
Minute ventilation (L)	10.9 (2.2)	10.7 (2.3)	10.7 (2.2)	10.2 (2.3)	0.5	*0.262*
Peak airway pressure (cmH_2_O)	27 (4)	26 (4)	28 (7)	35 (7)	-9	***<0.001***
Mean airway pressure (cmH_2_O)	19 (4)	18 (4)	20 (4)	22 (5)	-3	***0.021***
Dead space/tidal volume ratio	0.61 (0.14)	0.62 (0.14)	0.58 (0.15)	0.52 (0.14)	0.10	***0.001***
pH	7.37 (0.12)	7.37 (0.14)	7.38 (0.13)	7.41 (0.12)	-0.04	***0.001***
PaCO_2 _(mmHg)	47 (13)	49 (15)	48 (14)	43 (12)	6	***0.002***
PaO_2 _(mmHg)	124 (49)	131 (58)	120 (38)	117 (47)	14	***0.020***^a^
Heart rate (1/min)	92 (16)	92 (15)	91 (17)	93 (19)	-1	*0.521*
Mean arterial pressure (mmHg)	78 (10)	75 (13)	78 (14)	77 (12)	-2	*0.514*
Cardiac index (L.min^-1^.m^-2^)	3.8 (1.3)	3.9 (1.2)	3.9 (1.2)	3.5 (1.1)	0.4	***0.012***
Central venous pressure (mmHg)	12 (5)	11 (5)	12 (4)	12 (5)	-1	*0.176*
Systemic vascular resistance index (dyne.sec.cm^-5^.m^-2^)	1494 (500)	1374 (312)	1455(432)	1572 (469)	-198	***0.008***
Oxygen delivery index (mL.min^-1^.m^-2^)	506 (181)	521 (187)	509 (145)	463 (146)	58	***0.002***
Oxygen consumption index (mL.min^-1^.m^-2^)	171 (55)	172 (43)	182 (50)	186 (43)	-14	*0.239*
Carbon dioxide production index (mL.min^-1^.m^-2^)	115 (24)	110 (24)	120 (24)	122 (24)	-12	***0.002***
Oxygen extraction ratio	0.37 (0.13)	0.36 (0.08)	0.37 (11)	0.44 (0.11)	-0.07	***0.027***
Central venous saturation (%)	77 (9)	77 (8)	78 (8)	74 (9)	2	*0.120*
Arterial lactate (mmol/L)	1.6 (0.9)	1.5 (0.8)	1.5 (0.8)	1.6 (0.9)	-0.1	***0.028***

Reported is also the within-patient difference between the value after low TV and the value after high TV ('Low *vs*. High TV'), with *P *value from the analysis of variance adjusted for baseline. In bold are *P *values < 0.05.

^a^Analysis performed on log-transformed variable.

**Figure 2:**
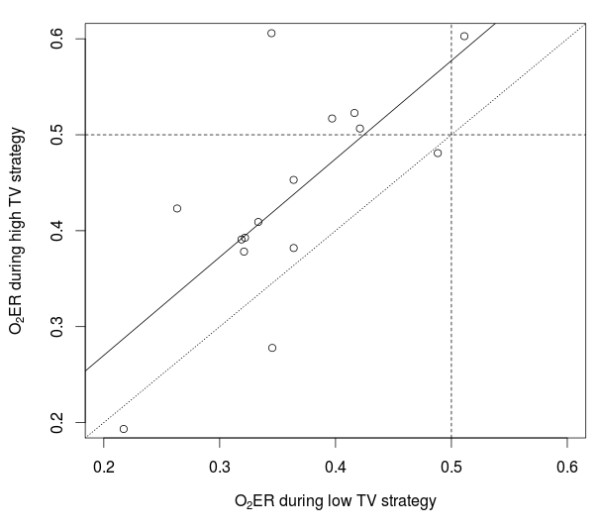
**Relationship between oxygen extraction ratio with low and high tidal volume (TV) strategies**. See text for details. Correlation between O_2_ER during high TV strategy and O_2_ER during low TV strategy: r = 0.68 (*P *= 0.003). Linear regression (solid line): O_2_ER during high TV strategy = 0.07 + O_2_ER during low TV strategy. Dotted line: identity line. O_2_ER: oxygen extraction ratio; TV: tidal volume.

Table 2 shows the effects of the two TV strategies on respiratory and cardiovascular parameters, with *P *values obtained from the analysis of variance adjusted for baseline values. We found no statistically significant evidence for period effects or carry-over effects. Low TV was associated with lower peak and mean airway pressure, slightly increased PaCO_2 _and PaO_2_, and decreased pH compared with high TV strategy. Minute ventilation remained constant during both ventilation strategies, whereas the dead space/TV ratio was higher during low compared with high TV strategy. Low TV strategy increased cardiac index and oxygen delivery and decreased the oxygen extraction ratio compared with high TV strategy, while heart rate and blood arterial pressure were similar in the two experimental groups. Carbon dioxide production was lower during low TV than during high TV ventilation. Oxygen consumption was obtained in 15 out of 16 patients, so oxygen extraction ratio was calculated in 15 subjects. Low TV was associated with decreased oxygen extraction ratio compared with high TV strategy (Table 2), with only one patient (7%) with an oxygen extraction ratio >0.5 during low TV, as opposed to five patients (33%) with oxygen extraction ratio ≥0.5 during high TV strategy (Figure 2). Comparing low with high TV strategy, the amount of the increase in TV was not associated with the magnitude of the reduction in cardiac index (*P *= 0.63). Similarly, the magnitude of the increase in peak airway pressure was not related to the extent of the change in cardiac index (*P *= 0.62). PaCO_2 _changes were positively associated with cardiac index variations (*P *= 0.004, r^2 ^= 0.41) and inversely associated with changes in the systemic vascular resistance index (*P *= 0.01, r^2 ^= 0.32).

The increase of cardiac index associated with low TV was predicted by the increase of PaCO_2_, with an area under ROC curve of 0.73 (95% confidence interval (CI): 0.57-0.88). For a threshold of 7 mmHg increase in PaCO_2_, specificity was 100% (95% CI: 48%-100%) and sensitivity 45% (95% CI: 17%-77%).

During low TV ventilation, 20% (IQR 17-28%) of minute ventilation resulted from spontaneous respiratory activity compared with 7% (IQR 0-19%) during high TV ventilation, although the difference was not statistically different (*P *= 0.27).

## Discussion

The present study showed that low TV increases cardiac index and oxygen delivery in ARDS patients compared to high TV strategy, when minute ventilation is maintained constant. Our findings suggest that this effect may be due to a slight increase in PaCO_2 _which induces reduction in systemic vascular resistance. The reduction in oxygen delivery during high TV strategy might be critical in those patients increasing oxygen extraction ratio >50%.

The effect of different TVs on cardiac output and oxygen delivery was investigated in previous studies with conflicting results. The earlier study [9] did not really test the effect of low TV ventilation because normal TV was 14 mL/kg whereas low TV was 11 mL/kg of actual body weight. Indeed this low TV was similar to the current definition of high TV. In this study cardiac index increased during 'low' TV ventilation compared to ventilation with TV 14 mL/kg. Three successive studies compared TVs more similar to current definition of low and high TV ventilation (6-8 mL/kg *vs*. 9-13 mL/kg) but did not modify respiratory rate at different TVs [10,12,13]. The consequence was that minute ventilation was dramatically decreased and PaCO_2 _showed large increase during low as opposed to high TV ventilation. These three studies showed an increase of cardiac index during low compared to high TV. Nevertheless the choice to maintain constant respiratory rate despite TV change is not consistent with the modern concept of low TV ventilation that is delivered at higher respiratory rate than high TV ventilation to maintain constant minute ventilation with minimal PaCO_2 _increase [1]. The only study, by Kiiski et al., investigating the effect of different TVs at constant minute ventilation in ARDS patients did not show any difference in cardiac output between low and high TV strategies [11]. The reported PaCO_2 _increase associated with lower TV ventilation was consistent with our findings (3.75 and 6 mmHg, respectively), and in both studies the PaCO_2 _increase was associated with an increase in dead space/TV ratio. Despite these similarities, there are both physiological and methodological reasons which could explain the different findings between Kiiski's and our study. While in Kiiski's study all patients were deeply sedated and paralyzed to prevent any sign of spontaneous respiratory activity, we maintained active respiratory activity whenever possible if patient-ventilator synchrony was present. Differences in sedation level between the two studies might explain the discrepancy in the response of cardiac index to PaCO_2 _changes, through differences in sympathetic stimulation. Moreover, the mere presence of spontaneous respiratory activity can increase cardiac output as opposed to muscle paralysis in ARDS patients with constant minute ventilation [17]. In our study, spontaneous respiratory activity disappeared in six patients (38%) during high TV strategy, and this might have had some impact on their cardiovascular function.

The results of our investigation are consistent with the current concept of low and high TV strategies [1], and with the recognized role of spontaneous respiratory activity during assist-control ventilation to prevent ventilator-induced diaphragm dysfunction [18]. Most patients showed active interaction with mechanical ventilatory support, thus precluding any meaningful interpretation of airway plateau pressure. Nevertheless, peak airway pressure is expected to be close to plateau airway pressure, due to the low or null airflow at the end of inspiration which characterizes the Pressure-Regulated Volume-Controlled Ventilation. This mode of ventilation works by applying a constant airway pressure during the inspiratory time to obtain the set tidal volume: the applied pressure is regulated breath by breath to maintain the target volume. As in every assist-control mode, respiratory rate can be increased above the set respiratory rate if the inspiratory trigger is activated. In patients with sustained inspiratory activity during the inspiratory time, TV can be higher than the set TV without any increase in airway pressure: the driving pressure is increased by the decrease of the pressure within the lungs due to spontaneous inspiratory activity. Nevertheless the increase of TV is self-limiting since, whenever TV is above the target, the inspiratory pressure is decreased in the following breaths to obtain again the set TV. Therefore, in this mode of ventilation airway pressure changes slowly breath by breath, and both peak and mean airway pressure well describe the applied pressure.

Oxygen extraction ratio was lower during low TV than during high TV strategy. Previous laboratory and clinical investigations showed that a critical oxygen extraction ratio >50% is associated with the development of organ dysfunction and predicts outcome [19-21]. During low TV ventilation only one patient (7%) had critical oxygen extraction ratio, whereas during high TV strategy five out 15 patients (33%) showed critical oxygen extraction ratio. The small, but significant, decrease of arterial lactate associated with low TV ventilation is consistent with these findings. Although the evaluation of the impact of oxygen delivery changes on organ failure and survival was beyond the remit of our study, this finding should be taken into account because a similar condition lasting a sufficient time could worsen organ function and patient outcome. The reduction of critical oxygen delivery episodes could constitute an additional beneficial effect of low as opposed to high TV strategy. On the other hand, an increase of fluid administration to correct this pattern could have detrimental effects on prognosis in patients with ARDS [22,23].

Interestingly, CO_2 _production showed a small, even though statistically significant, reduction during low TV compared with high TV ventilation strategy, in agreement with previous findings from Kiiski et al. [11]. In non-steady-state conditions, the elimination of CO_2 _from the alveoli depends on both CO_2 _metabolic production and change of body CO_2 _pool [11]. Therefore the observed difference in CO_2 _production is more likely to reflect the ongoing stabilization of body CO_2 _pool, since during high TV ventilation more CO_2 _is eliminated as a consequence of the progressive decrease of CO_2 _storage in the body.

Changes in airway pressure and TV were not quantitatively related to cardiac index changes. On the contrary, the extent of PaCO_2 _variation was related to cardiac index difference between low and high TV ventilation. This finding is consistent with a previous observation that showed cardiac index increased when PaCO_2 _was raised in response to instrumental dead space changes at constant ventilation [24]. In our study the amount of PaCO_2 _difference between TVs was on average 6 mmHg and it is the expected mean difference between high and low TV strategy [1]. Both present and previous data support the concept that a decrease in systemic vascular resistance induced by mild hypercapnia could increase cardiac output. This is confirmed by the strong predictive value of PaCO_2 _increase of 7 mmHg or higher, with such a change being always associated with cardiac index increase in our patients (specificity 100%). The relationship between changes of PaCO_2 _and cardiac index observed in our study, although consistent with current physiological and clinical knowledge, is derived from a post-hoc analysis and can explain less than half of the variation of cardiac index (r^2 ^= 0.4). Therefore we cannot draw definitive conclusions, and it is likely that other mechanisms in addition to hypercapnia might have contributed to the increase in cardiac index associated with low TV ventilation.

Low TV improved PaO_2 _when compared to high TV strategy. This result can be explained by at least three different physiological mechanisms leading to an improvement in the ventilation-perfusion mismatch, a well-recognized cause of hypoxemia in ARDS patients [25]. First, low TV compared to high TV strategy increases spontaneous respiratory activity improving ventilation in juxta-diaphragmatic pulmonary zones [26]. This effect is sufficient to improve ventilation-perfusion distribution in ARDS patients [17]. Second, ARDS lungs do not have homogeneous distribution of ventilation and therefore have different time constants [27]. Fast alveoli are exposed to a greater risk of overdistension than slow alveoli: higher tidal alveolar pressure could locally compress pulmonary capillaries (28) averting blood flow from these well-ventilated areas toward slow alveoli that are less ventilated. The consequence should be an increase of ventilation-perfusion mismatch that could be minimized by TV reduction. Third, mild hypercapnia, induced by low TV strategy, could increase pulmonary vasoconstriction [28] and thereby improving ventilation-perfusion distribution [29,30].

### Study limitations

The impact of TV on stroke volume depends on intravascular volume [5,6,31]. Our study, like previous similar studies [9-13], enrolled only patients with invasive monitoring of cardiac output after obtaining stable cardiovascular state. Consequently patients were normotensive, had normal-to-high values of cardiac output and did not need changes in cardiovascular support during the study. Accordingly our study results are valid only for stable patients after cardiovascular optimization.

Low and high TV strategies differ both for the set TV and for the consequent changes on some physiological variables, including respiratory rate, patient inspiratory effort, PaCO_2_. Our study was designed to evaluate the overall effect of each ventilatory strategy on cardiovascular function, as it occurs in daily clinical practice, and therefore could not disentangle the effects of the different ventilatory parameters.

In our study low TV strategy was more similar to baseline ventilation than high TV strategy: consequently baseline intravascular volume could be more appropriate for low TV than for high TV strategy. Then we cannot exclude that high TV strategy could require additional fluid administration to restore stroke volume and oxygen delivery [5-8]. Nevertheless, the increase of fluid administration and positive fluid balance could transiently improve hemodynamics but are associated to the increase in ICU length of stay and mortality [22,23].

It is important to consider that our results were obtained in patients with PaCO_2 _and PEEP level slightly different from those reported in the ARDSNet trial [1]. In the ARDSNet study, the respiratory rate was targeted to keep pH within a range (7.3-7.45), and not to keep constant minute ventilation. As a consequence, in the ARDSNet trial the average PaCO_2 _during the first day was normal (40 mmHg) during low TV ventilation, whereas it was on average 49 mmHg in our study. This may differently influence the hemodynamic response and respiratory drive, as discussed above. Nevertheless, the average pH during low TV ventilation in the ARDSNet trial was similar to that in our study (7.38 and 7.37, respectively), and this minimizes the possible impact of the observed difference in PaCO_2 _between the two studies. In the ARDSNet study PEEP was set according to a PEEP/F_I_O_2 _table whereas we set PEEP according to respiratory mechanics. As a result, the average PEEP value was lower in the ARDSNet trial than in our study (about 9 cmH_2_O *vs*. 14 cmH_2_O, respectively); moreover in the ARDSNet trial, PEEP was slightly higher during low than high TV ventilation (9.4 *vs*. 8.6 cmH_2_O, respectively) because PaO_2 _was initially lower during 6 mL/kg TV than during 12 mL/kg TV. The fact that we enrolled only fluid-resuscitated patients with stable cardiovascular function should exclude that such PEEP difference could influence the effect of low and high TV strategy on cardiac output. Indeed the increase of PEEP from 10 to 15 cmH_2_O did not modify cardiac transmural filling pressure and cardiac output in volume-loaded condition [32]. Moreover, PEEP level around 15 cmH_2_O is usual when PEEP is set on mechanical properties of respiratory system and this approach seems to maximize the beneficial effect of protective ventilation (2,33,34). Furthermore, high PEEP level (about 15 cmH_2_O) is associated with reduced mortality when compared to low PEEP level (about 9 cmH_2_O) in patient with moderate-to-severe ARDS (35).

Finally, cardiac output can be affected by the patient's inspiratory effort. We did not measure esophageal pressure to estimate the level of spontaneous respiratory activity, and we therefore cannot exclude that spontaneous breathing activity could contribute to the increase of cardiac output observed during low volume strategy. Therefore our results should be taken into account mainly for patients with assist-control ventilation when spontaneous breathing activity is allowed.

## Conclusions

Our findings support the hypothesis that low TV ventilation, as currently defined, improves cardiac index and oxygen delivery when compared to high TV strategy. This effect could be clinically relevant in some patients and could represent an additional favorable effect of TV reduction. In other words, low tidal volume ventilation could be protective also for the cardiovascular system and not only for the lung. Cardiac index increase may be mediated by mild respiratory acidosis which develops during TV decrease.

## Key messages

• Low tidal volume ventilation strategy improves cardiac index and oxygen delivery when compared to high tidal volume strategy in ARDS patients.

• Critically high values of oxygen extraction ratio can be observed during high but not during low tidal volume ventilation strategy.

• A mild-to-moderate carbon dioxide increase is an element of low tidal volume strategy in clinical practice.

• The magnitude of the hemodynamic improvement associated with low tidal volume ventilation is related to the magnitude of carbon dioxide increase, but not to that of tidal volume or airway pressure variations.

• The improvement of hemodynamics could represent an additional favorable effect of low tidal volume respect to high tidal volume strategy.

## Abbreviations

ALI: acute lung injury; ARDS: acute respiratory distress syndrome; CI: confidence interval; IQR: interquartile range; PiCCO: Pulse-induced Continuous Cardiac Output; PEEP: positive end-expiratory pressure; ROC: receiver operating characteristic; TV: tidal volume.

## Competing interests

The authors declare that they have no competing interests.

## Authors' contributions

GN conceived of the study, participated in its design, conducted the study, performed the statistical analyses, and wrote the manuscript. CM performed the statistical analyses and wrote the manuscript. AR participated in the design of the study and conducted the study. PF helped to conduct the study. CRM helped to conduct the study. CDF helped to conduct the study. AB participated in the design of the study.

All authors read and approved the final manuscript.
